# Supplementary figures and images for: Information sharing in high-dimensional gene expression data for improved parameter estimation in concentration-response modelling
Source: PLoS One. 2023 Oct 20;18(10):e0293180. doi: 10.1371/journal.pone.0293180 (PMC10588876; doi:10.1371/journal.pone.0293180)

Frequency

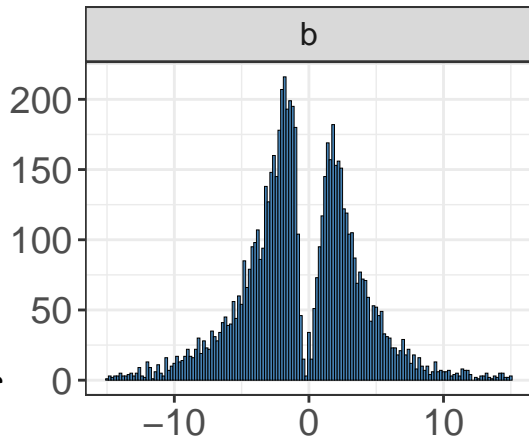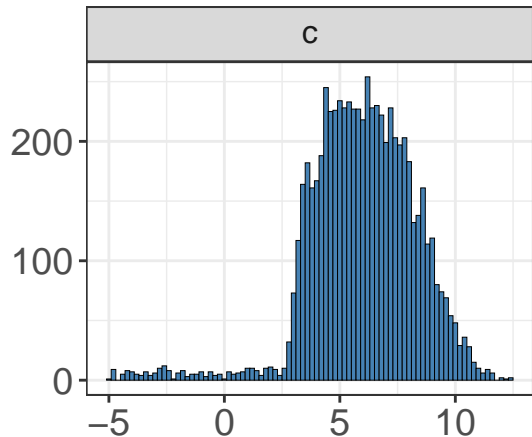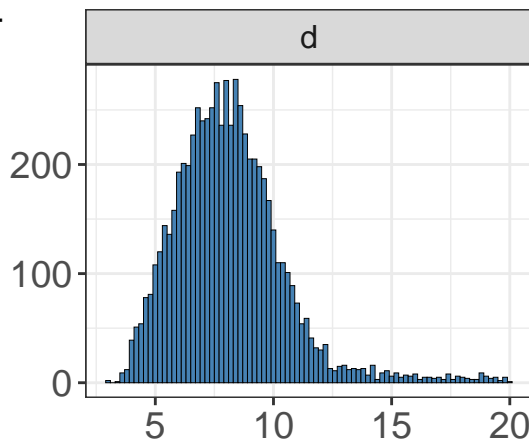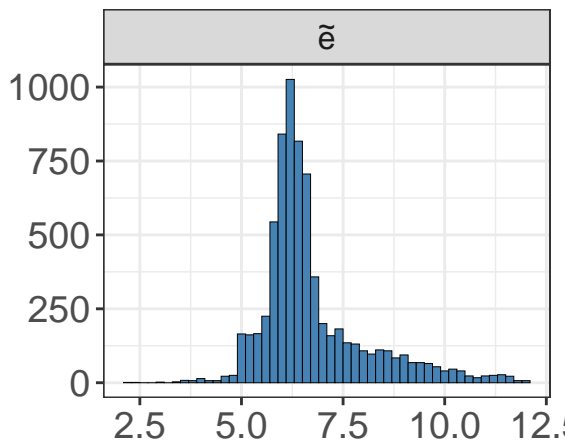

Parameter Value

Supplement: S1 Fig — Estimates of the four parameters b,c,d,e˜ of the 4pLL model, fitted to the 7191 genes selected from the VPA dataset, are shown by histograms. (PDF) [file pone.0293180.s001.pdf]

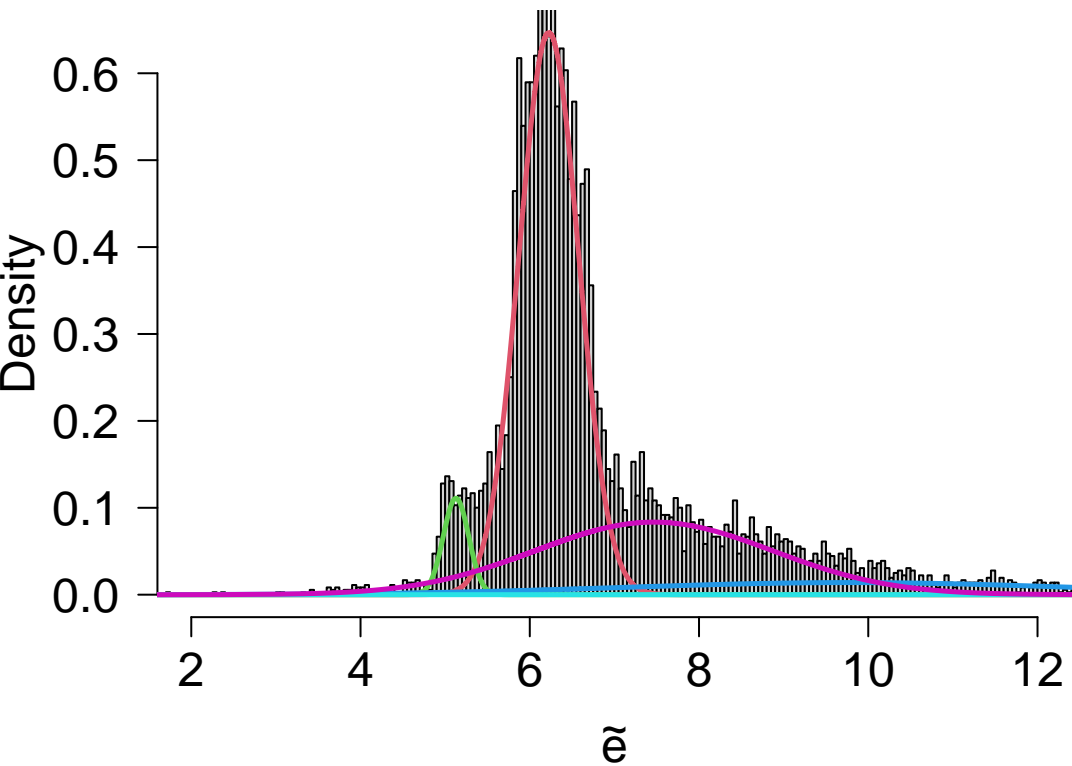

Supplement: S2 Fig — The corresponding parameter estimates are summarized in Table 2, where the first component corresponds to the red curve, the second component to the green curve, the third component to the blue curve, the fourth component corresponds to the turquoise curve, and the fifth component corresponds to the purple curve. (PDF) [file pone.0293180.s002.pdf]

**A)**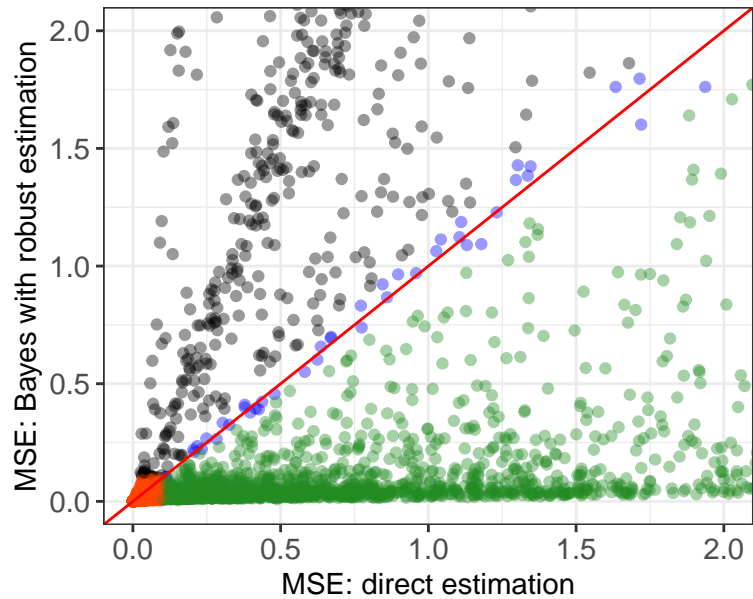**B)**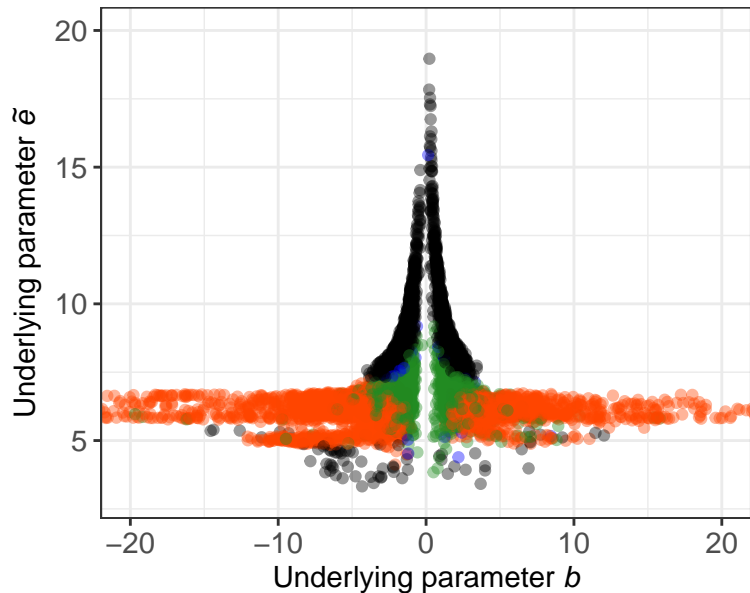

Supplement: S3 Fig — The resulting values of the MSE are colored according to the comparative performance of the two approaches. The underlying parameter values are colored in the same way. (PDF) [file pone.0293180.s003.pdf]

**A)**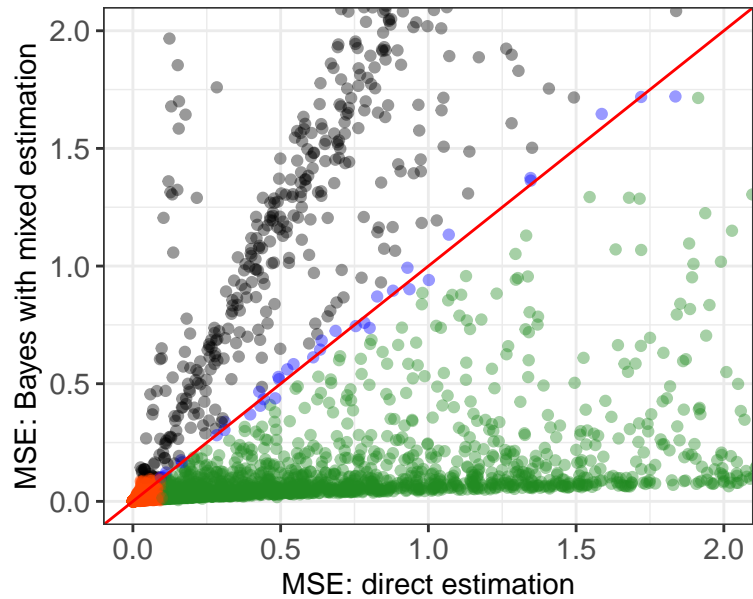**B)**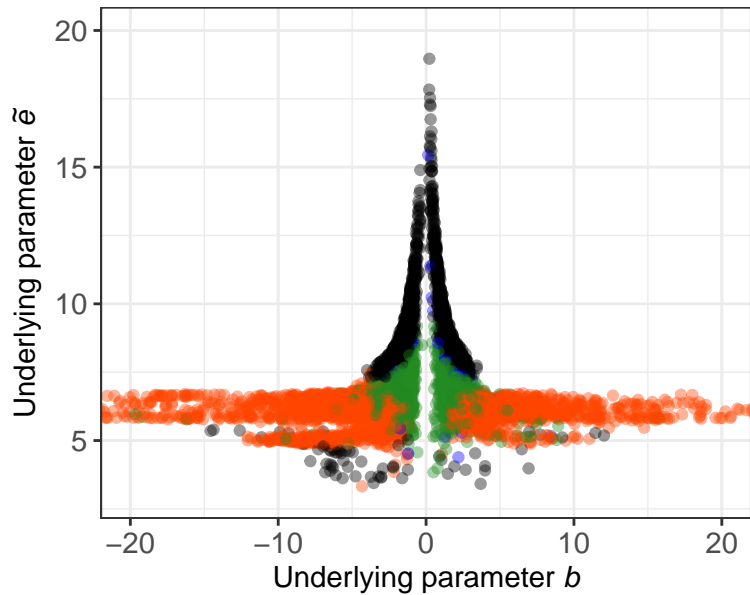

Supplement: S4 Fig — The resulting values of the MSE are colored according to the comparative performance of the two approaches. The underlying parameter values are colored in the same way. (PDF) [file pone.0293180.s004.pdf]

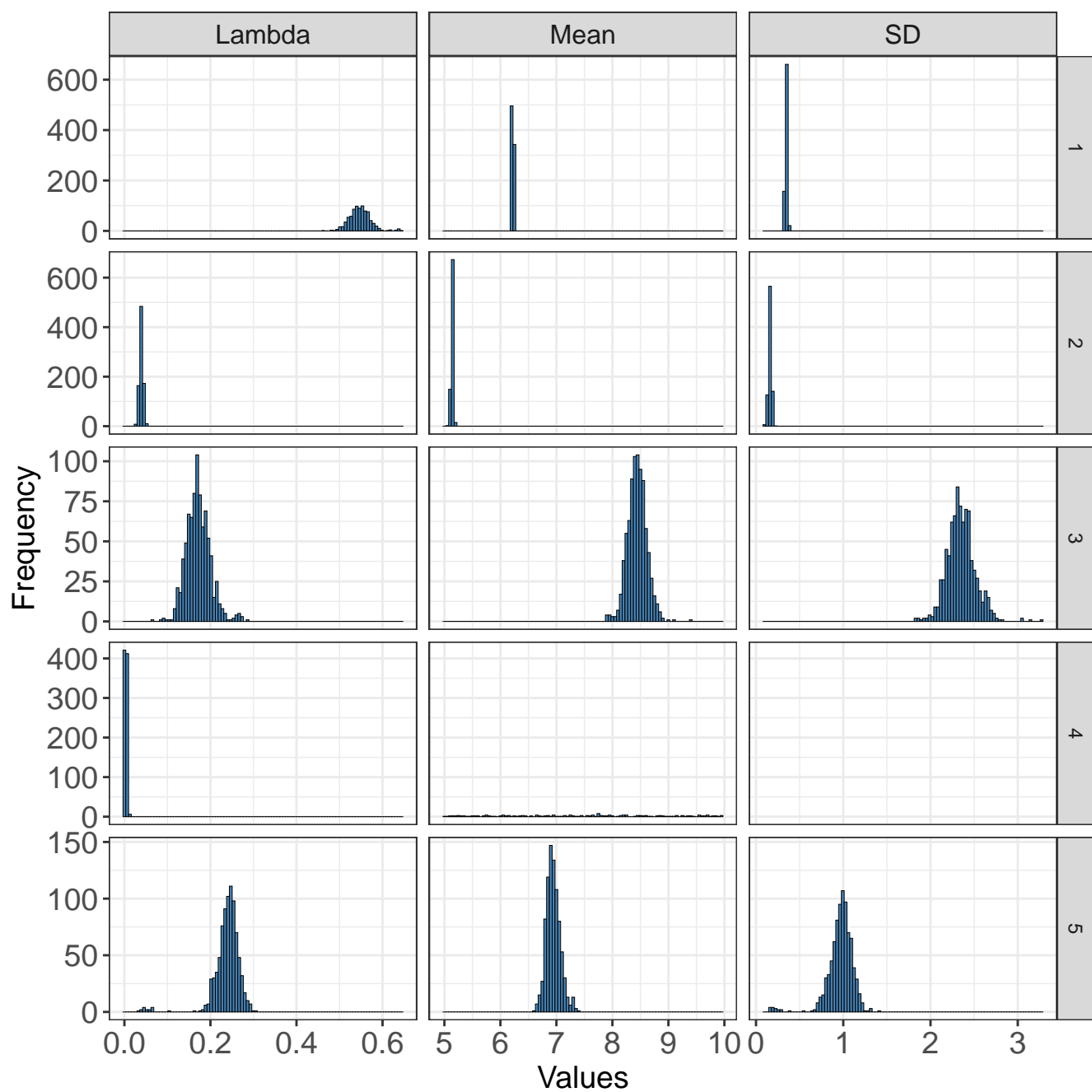

Supplement: S6 Fig — The five rows show the individual mixture components, and the columns the mixing parameter λ (left), the prior mean (middle) and the prior standard deviation (right) of the respective mixture component. (PDF) [file pone.0293180.s006.pdf]
