# Supplementary material for: Information sharing in high-dimensional gene expression data for improved parameter estimation in concentration-response modelling
Source: PLoS One. 2023 Oct 20;18(10):e0293180. doi: 10.1371/journal.pone.0293180 (PMC10588876; doi:10.1371/journal.pone.0293180)
Supplement: S5 Fig — The two histograms on the left (A) show the values for the prior mean of the normal distribution, the two histograms on the right (B) show the values for the prior standard deviation. The parameters are estimated via ML estimation (top) or via robust estimation (bottom). (PDF) [file pone.0293180.s005.pdf]

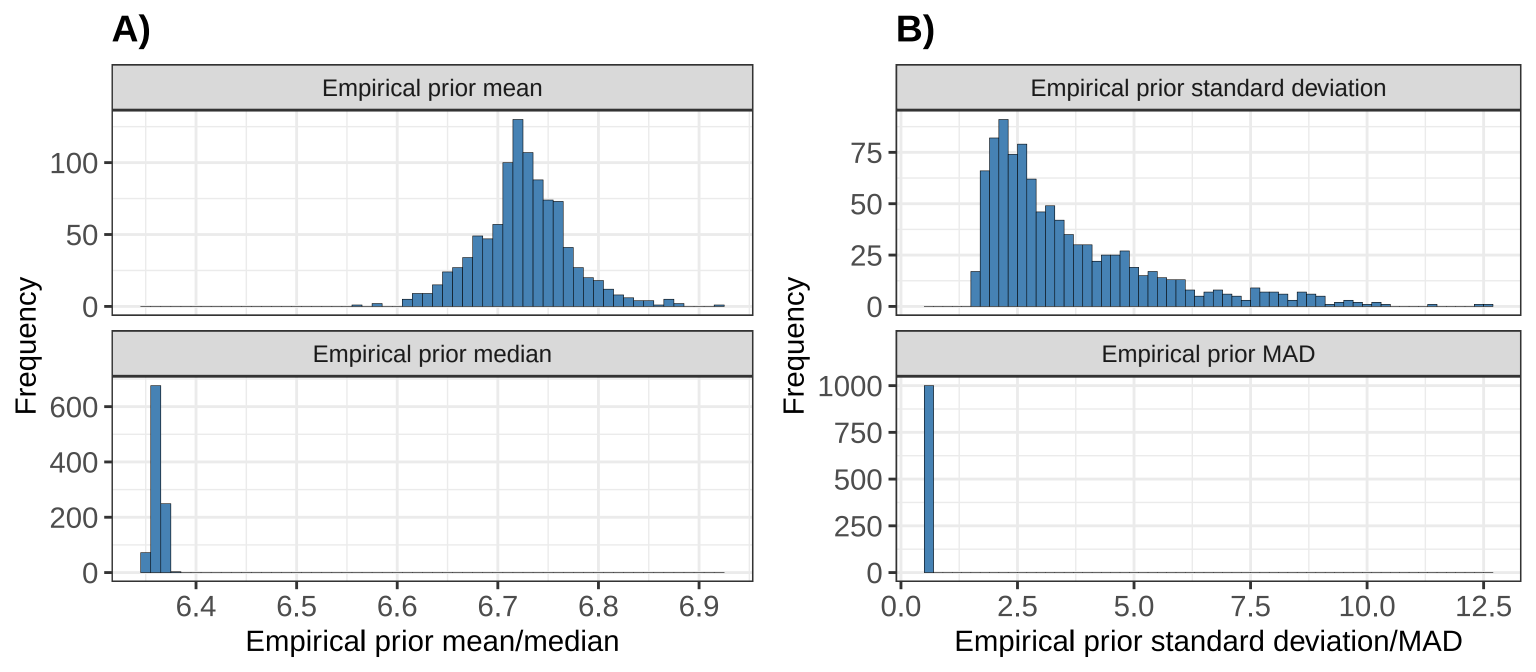
A)
B)
Empirical prior mean
Empirical prior median
Empirical prior standard deviation
Empirical prior MAD
Frequency
100
50
0
600
400
200
75
25
1000
750
500
250
6.4
6.5
6.6
6.7
6.8
6.9
0.0
2.5
5.0
7.5
10.0
12.5
Empirical prior mean/median
Empirical prior standard deviation/MAD
