# Supplementary material for: Information sharing in high-dimensional gene expression data for improved parameter estimation in concentration-response modelling
Source: PLoS One. 2023 Oct 20;18(10):e0293180. doi: 10.1371/journal.pone.0293180 (PMC10588876; doi:10.1371/journal.pone.0293180)
Supplement: Attachment — Submitted filename: Response.pdf [file pone.0293180.s007.pdf]

Response

The paper titled "Information sharing in high-dimensional gene expression data for improved parameter estimation in concentration-response modelling" utilizes a four-parameter logistic function to model toxicological concentration-response, a model that was previously proposed [1,2,3]. The novelty of this work lies in the application of a Bayesian approach to obtain probabilities (posterior distributions fed by previous information). This approach considers the alert concentration EC50 as a parameter. Ultimately, the results are compared with those obtained using other three different approaches.

Considering that PLOS ONE objectively focuses on the technical aspects of a study rather than subjective evaluations, and after having read the paper I recommend rejecting the current manuscript. My reasons for this recommendation are explained below.

- From the Problem Conceptualization Perspective:

  There were so many terms that are ambiguous and make the text difficult to understand as they are not properly defined. Specially in the abstract and the introduction sections, the problem is not well contextualized, especially considering that "dose-response" is employed in various contexts.

  When discussing toxicological concentrations, it is crucial to clearly specify the biological system right from the beginning, along with the associated components that we intend to evaluate. The values described as EC50 and ED90 (line 35) are complex to interpret for individuals who are not familiar with chemical compounds or the health sector. Considering that one of the most cited references in the manuscript is [2], a book titled "Dose-Response Analysis Using R," this leads to vastly different terms that, without being specifically defined and delimited, become more difficult to follow. Other terms such as MSE should be defined from the outset as mean squared error.

- From the method:

  The four-parameter logistic function (Eq. 1) has been used to model effects across different concentration ranges. In my understanding, the novelty of this manuscript lies in the application of a Bayesian approach to obtain probabilities (posterior distributions fed by previous information). The Bayesian interpretation is straightforward. Revisiting the formula with consideration of your variables I may have:

$$p(\mu|y) = \frac{p(\mu|y)\, p(\mu)}{p(y)}$$

a) The prior $p(\mu)$, initially defined as a normal distribution, is later replaced with a more flexible one involving five parameters. This transition is challenging to follow, especially considering your earlier mention that the parameter vector comprises only four parameters. $\varphi \coloneqq \{b, c, d, e\}$
b) The likelihood $p(\mu|y)$, the information about μ contained in y. I intuitively guess is related with $f(x, \varphi)$, (although not explicitly mentioned), since you are employing this logistic function to model the concentrations.
c) $p(y)$ a normalising constant independent of μ.

For Bayesian inference, it is important to present the kernel or the form of the posterior distribution in terms of the parameters of your model (not necessarily the normalization coefficients). This step is crucial for validating the simulations that can be executed with any software or package. Unfortunately, the study section only provides a description of the programming language and the package used. Additionally, I cannot find information on how sensitive the data is to the prior, other than the direct assignment that the prior follows a Gaussian distribution function.

The results are presented as fitted values (estimated values) described in Tables 2 and 3, and they are compared among direct fit, robust Bayes, and mixing distribution Bayes methods. Attached are figures that illustrate the execution of multiple simulations. I can observe probabilities for the posterior distributions in the Supporting Information; however, I cannot see or visualize the model considering the obtained parameters in relation to the used data. For instance, after Eq(1), it is mentioned that variable $\tilde{e}$ is related to EC50 (Effective Concentration to 50%). While other values are obtained, their level of significance is not specified. Moreover, simply listing the results without providing precision or indicating whether their posterior convergence is achieved leaves some uncertainty.

[1] Cedergreen, N., Ritz, C., & Streibig, J. C. (2005). Improved empirical models describing hormesis. *Environmental Toxicology and Chemistry: An International Journal*, *24*(12), 3166-3172.

[2] Knezevic, S. Z., Datta, A., Scott, J., & Porpiglia, P. J. (2009). Dose–response curves of KIH-485 for preemergence weed control in corn. *Weed Technology*, *23*(1), 34-39.

[3] Altop, E. K., Mennan, H., Streibig, J. C., Budak, U., & Ritz, C. (2014). Detecting ALS and ACCase herbicide tolerant accession of Echinochloa oryzoides (Ard.) Fritsch. in rice (Oryza sativa L.) fields. *Crop Protection*, *65*, 202-206.
