# Supplementary material for: Information sharing in high-dimensional gene expression data for improved parameter estimation in concentration-response modelling
Source: PLoS One. 2023 Oct 20;18(10):e0293180. doi: 10.1371/journal.pone.0293180 (PMC10588876; doi:10.1371/journal.pone.0293180)
Supplement: Attachment — Submitted filename: Response_InformationSharing.pdf [file pone.0293180.s008.pdf]

Answer to referee reports for the manuscript:

Franziska Kappenberg, Jörg Rahnenführer

Information sharing in high-dimensional gene expression data for improved parameter estimation in concentration-response modelling

---

Dear Editor, dear Reviewers,

thank you very much for the helpful comments. We have carefully addressed all points. Our changes are indicated in the new version of the manuscript in red.

In the point-by-point letter the comments of the reviewers are printed in bold, followed by our answers. The individual points raised in the separate file by Reviewer #3 are pasted into this letter separately, followed by our answers and references to changes in the manuscript.

We are looking forward to your response.

Best regards
Franziska Kappenberg and Jörg Rahnenführer

Point-by point letter:

Reviewer #1:

In the current era, simulation studies are growing in the field of biological studies. Yet, it’s still a growing field. In the same sense, the presented paper proposes a new method to improve the estimation of minimal toxicity. The authors used an approach where information is shared across genes which allows the relaxation of common parameters and hence allows some improvements in the statistical analyses. Empirical Bayes approaches were used to carry out the study. Results were clearly detailed and explained. The overall notion shows significant merit in the application of robust Bayes and mixing distribution Bayes approaches for acquiring more information from certain data. Indeed, this could be useful for several biological models.

Thank you for this positive assessment of the work.

Reviewer #2:

Your approach to information sharing in high-dimensional gene expression data is both innovative and insightful. The utilization of concentration-response

modeling, coupled with the incorporation of information sharing techniques, showcases your expertise in handling complex biological datasets. The results you have obtained demonstrate the potential for improved parameter estimation, which holds promising implications for advancing our understanding of gene expression patterns and their regulatory mechanisms.

I commend your meticulous data analysis and the clarity with which you have presented your findings. Your attention to detail and rigorous methodology inspire confidence in the validity and reliability of your research outcomes. Furthermore, your ability to effectively communicate complex concepts ensures that your work can be understood and appreciated by both fellow researchers and non-experts alike.

In addition to the scientific rigor, your work also exemplifies your passion for the subject matter. It is evident that you possess a genuine curiosity for unraveling the intricacies of gene expression, and this enthusiasm shines through in every aspect of your research.

Thank you for these very positive and encouraging words about our work!

Reviewer #3:

The paper titled "Information sharing in high-dimensional gene expression data for improved parameter estimation in concentration-response modelling" utilizes a four-parameter logistic function to model toxicological concentration-response, a model that was previously proposed. The novelty of this work lies in the application of a Bayesian approach to obtain probabilities (posterior distributions fed by previous information). This approach considers the alert concentration EC50 as a parameter. Ultimately, the results are compared with those obtained using other three different approaches.

Considering that PLOS ONE objectively focuses on the technical aspects of a study rather than subjective evaluations, and after having read the paper I recommend rejecting the current manuscript. My reasons for this recommendation are attached in a file.

We included the remarks from the file in this letter and answer the points individually.

Response

The paper titled "Information sharing in high-dimensional gene expression data for improved parameter estimation in concentration-response modelling" utilizes a four-parameter logistic function to model toxicological concentration-response, a model that was previously proposed [1,2,3]. The novelty of this work lies in the application of a Bayesian approach to obtain probabilities (posterior distributions fed by previous information). This approach considers the alert concentration EC50 as a parameter. Ultimately, the results are compared with those obtained using other three different approaches.

Considering that PLOS ONE objectively focuses on the technical aspects of a study rather than subjective evaluations, and after having read the paper I recommend rejecting the current manuscript. My reasons for this recommendation are explained below.

[1] Cedergreen, N., Ritz, C., & Streibig, J. C. (2005). Improved empirical models describing hormesis. *Environmental Toxicology and Chemistry: An International Journal*, *24*(12), 3166-3172.

[2] Knezevic, S. Z., Datta, A., Scott, J., & Porpiglia, P. J. (2009). Dose–response curves of KIH-485 for preemergence weed control in corn. *Weed Technology*, *23*(1), 34-39.

[3] Altop, E. K., Mennan, H., Streibig, J. C., Budak, U., & Ritz, C. (2014). Detecting ALS and ACCase herbicide tolerant accession of Echinochloa oryzoides (Ard.) Fritsch. in rice (Oryza sativa L.) fields. *Crop Protection*, *65*, 202-206.

- From the Problem Conceptualization Perspective:

  There were so many terms that are ambiguous and make the text difficult to understand as they are not properly defined. Specially in the abstract and the introduction sections, the problem is not well contextualized, especially considering that "dose-response" is employed in various contexts.

  When discussing toxicological concentrations, it is crucial to clearly specify the biological system right from the beginning, along with the associated components that we intend to evaluate. The values described as EC50 and ED90 (line 35) are complex to interpret for individuals who are not familiar with chemical compounds or the health sector. Considering that one of the most cited references in the manuscript is [2], a book titled "Dose-Response Analysis Using R," this leads to vastly different terms that, without being specifically defined and delimited, become more difficult to follow. Other terms such as MSE should be defined from the outset as mean squared error.

The context of the problem and the proposed method is specifically identified in the abstract and the Section Introduction. In the corresponding first sentences, toxicological

research in the context of gene expression analysis is identified as application for the proposed method.

Regarding the understanding and interpretation of EC values, we added additional explanatory sentences and paragraphs in three positions of the Section Introduction.

First, a general definition of EC values is given with the following statement:

"For cell viability experiments, where the measured response is given by some percentage, EC values are typically calculated in an absolute way, i.e. as the concentration, where the fitted curve attains the specific pre-defined percentage. In applications such as gene expression data, where the responses themselves do not correspond to percentages, EC values are calculated in a relative way. In these cases, a certain percentage of the overall effect, i.e. the difference in the response values between the highest and lowest measured concentration (or between the two asymptotes of a fitted model), needs to be attained by the fitted curve."

Second, we added the following explanation to the formal explanation of the EC50 in our context in the Introduction:

"In the situation of gene expression as response data, as considered in this work, the EC50 is to be understood in a relative way."

Third, we provided context on the cited reference where a similar method has been used for ED90 values instead of EC50 values:

"Such an approach has been used before in [8], in a simpler way, where a mixture of two normal distributions was fitted for ED90 (here referring to doses instead of concentrations) values on log-scale, where the response value was given by the biomass of plants after treatment with different doses of a herbicide."

We also added the definition of the MSE as mean squared error to the abstract.

- From the method:

  The four-parameter logistic function (Eq. 1) has been used to model effects across different concentration ranges. In my understanding, the novelty of this manuscript lies in the application of a Bayesian approach to obtain probabilities (posterior distributions fed by previous information). The Bayesian interpretation is straightforward. Revisiting the formula with consideration of your variables I may have:

$$p(\mu|y) = \frac{p(\mu|y)\, p(\mu)}{p(y)}$$

There is a small typo in your formula, the correct formula is:

$$p(\mu|y) = \frac{p(y|\mu)p(\mu)}{p(y)}$$

Please note that, in our manuscript, instead of y, the letter x is used.

a) The prior $p(\mu)$, initially defined as a normal distribution, is later replaced with a more flexible one involving five parameters. This transition is challenging to follow, especially considering your earlier mention that the parameter vector comprises only four parameters. $\varphi := \{b, c, d, e\}$

The prior refers to the distribution of the parameter ẽ only, since this is the only parameter to which the Bayesian information sharing is applied. We added the sentence "Only this parameter from the 4pLL model is thus considered for the Bayesian information sharing" to the respective part of the Statistical Methods Section to underline this.

b) The likelihood $p(\mu|y)$, the information about μ contained in y. I intuitively guess is related with $f(x, \varphi)$, (although not explicitly mentioned), since you are employing this logistic function to model the concentrations.

The likelihood is not $p(\mu|y)$ (which corresponds to the posterior), but $p(y|\mu)$ (see general statement about the Bayes formula above). As stated in the previous paragraph, the Bayesian approach is not applied to the entire model $f(x, \varphi)$, but to the parameter ẽ only. So the likelihood is given by the formula $X|\mu \sim N(\mu, \sigma^2)$, as explicitly stated in the manuscript in the Section Statistical Methods.

c) $p(y)$ a normalising constant independent of μ.

This is correct, however it is not explicitly stated in the manuscript, since this is only a normalizing constant and does not need to be calculated for the Bayesian formula (which is typically formulated as a proportional relationship between posterior distribution on the one side and likelihood and prior on the other side).

For Bayesian inference, it is important to present the kernel or the form of the posterior distribution in terms of the parameters of your model (not necessarily the normalization coefficients). This step is crucial for validating the simulations that can be executed with any software or package. Unfortunately, the study section only provides a description of the programming language and the package used. Additionally, I cannot find information on how sensitive the data is to the prior, other than the direct assignment that the prior follows a Gaussian distribution function.

The posterior distribution is explicitly stated in Equation (2) of the manuscript. This Equation explicitly refers to the different parameters. All information about the formulas used for the simulation are thus explicitly given, and in addition the R-code for the simulation is made publicly available via github: https://github.com/FKappenberg/Paper-InformationSharingAcrossGenes. We added a reference to this repository in the Section Software of the manuscript.

We did not perform a sensitivity analysis with respect to the choice of the prior. However, the assumption of a Normal distribution is well-established in fitting of concentration-response curves, and the parameters directly stem from the data via our empirical Bayes approach, such that we believe our prior is suitable.

The results are presented as fitted values (estimated values) described in Tables 2 and 3, and they are compared among direct fit, robust Bayes, and mixing distribution Bayes methods. Attached are figures that illustrate the execution of multiple simulations. I can observe probabilities for the posterior distributions in the Supporting Information; however, I cannot see or visualize the model considering the obtained parameters in relation to the used data. For instance, after Eq(1), it is mentioned that variable ẽ is related to EC50 (Effective Concentration to 50%). While other values are obtained, their level of significance is not specified. Moreover, simply listing the results without providing precision or indicating whether their posterior convergence is achieved leaves some uncertainty.

Variable ẽ is the log-transformed EC50 parameter (see explanation directly below Equation (1) in the manuscript). Since the Bayesian information sharing approach is only conducted for this individual parameter and not for the entire model $f(x, \varphi)$, the results only refer to the estimation of this parameter ẽ.

The advantage of the Bayesian normal-normal model as employed in this work is the closed form of the posterior distribution (see Equation (2)). Thus, this Bayesian approach works without any sampling, such that no convergence needs to be achieved and the posterior can be calculated directly. Figure 5 shows coverage probabilities for the confidence intervals based on the direct estimation and for the credibility intervals based on the Bayesian approach. It can be observed that the general improvement in terms of the mean squared error does not come at a cost of lower coverage probability. Thus we believe that our presented results provide a full picture of the simulation results.
